# Archaea in Wastewater Treatment: Current Research and Emerging Technology

**DOI:** 10.1155/2018/6973294

**Published:** 2018-11-18

**Authors:** Jin Li, Rutao Liu, Yu Tao, Guangbin Li

**Affiliations:** ^1^School of Environmental Science and Engineering, Qingdao University, Qingdao 266071, China; ^2^School of Environmental Science and Engineering, Shandong University, Qingdao 266237, China; ^3^Department of Chemical Engineering, Imperial College London, London SW7 2AZ, UK; ^4^Department of Chemical and Environmental Engineering, University of Arizona, Tucson, AZ 85721, USA

Wastewater treatment is quite imperative for sustainable development and is critical for an ecosystem and for human health. Typically, wastewater is purified through multiple processes of microbial metabolisms. As a result, organic matters, ammonia, sulfate, and phosphate are either removed or transformed into other forms with lower harm to receiving aqua. We could not see a critical contribution of Archaea to wastewater treatment until recently when we learned that they are responsible for methane production, carbon mineralization, nitrification, and denitrification. Furthermore, a newly discovered denitrifying anaerobic methane oxidation process, through which methane is oxidized anaerobically, challenges the cliché concept of archaea involvement in wastewater treatment. To date, thousands of wastewater treatment facilities are confirmed to have an ecological and functional contribution by Archaea, which also benefits pollutant removal with low chemical and/or energy input.

Archaea-involved technology is essential for wastewater treatment by integrating energy production and resource recovery into a process for producing clean water. Archaea play important roles in converting pollutants into environmentally friendly materials. However, compared with bacteria that are widely studied in wastewater treatment systems, the characteristics and contributions of archaea are still not well known. For instance, ecological patterns of archaea in a complex wastewater microbiome are not fully understood, as well as the metabolisms of certain key archaea. For these reasons, we organized a special issue with a specific topic on why a comprehensive understanding of the identity, physiology, ecology, and population dynamics of archaea is urgently needed to improve wastewater treatment efficiency and process stability. It will be possible to find selective principles for regulating certain populations and managing a microbial community. It is entirely necessary to further study archaea in wastewater treatment. Such investigations can not only optimize the current wastewater treatment process but also innovate emerging technology.

Twelve papers, including both reviews and research articles, are selected in this Special Issue, covering the topics about the distributions and contributions of Archaea in wetlands, oilfield soil, and wastewater-treating bioreactors. Three review articles are highlighted in this Special Issue. One of the review papers focuses on the critical role of Archaea in bioremediation from halophilic hydrocarbon degradation to acidophilic hydrocarbon degradation in various environments such as oceans, soils, and acid mine drainage. Another article reviewed the characteristics and treatment of leachate, and more importantly, pointed out future directions for leachate research and development. The third review paper overviews the current knowledge on ammonium-oxidizing archaea and ammonium-oxidizing bacteria that are involved in wastewater treatment systems.

A wetland is an excellent combination of natural and engineered forms of wastewater treatment. Two research articles address bacterial and archaeal microbial community structures in constructed wetlands, with one focusing on microbiome differences between sediments and water, while another one focuses on microbial interactions in a pilot-scale wetland that treated saline wastewater from a land-based Atlantic salmon plant.

A microbial world in oilfield soil is unveiled in this Special Issue by two research articles, one focusing on physicochemical properties, contents of primary pollutants, and fungal diversity of an aged oil sludge-contaminated soil, while another one focuses on soil bacterial community diversity around an aging oil sludge in the Yellow River Delta of China.

Bioreactor microbiomes are the most popular topics in this Special Issue, including four research articles. An integrated biofilm-membrane bioreactor treating mustard tuber wastewater was reported with a particular focus on microbial mechanisms leading to membrane fouling. Another study focused on microbial community and the performance of an autohydrogenotrophic membrane biofilm reactor for removing nitrate from the wastewater with high sulfate concentrations. Anaerobic digestion reactors are also addressed in two research articles, with one focusing on a transitional role of hydrogen-producing acetogens and its application in bioaugmentation, and another one discussing the bacterial and archaeal roles in an integrated anaerobic fluidized-bed membrane bioreactor treating synthetic high-strength benzothiazole wastewater.

With rapid growth in biomonitoring tools, we have learned more details of archaea-involved bioprocesses than at any time in history. Following the publication of this current Special Issue, we expect more exciting breakthroughs of Archaea studies in wastewater treatment from theory improvement to technology innovation.

